# Repertory of the Back

**Published:** 1897-11

**Authors:** E. H. Wilsey

**Affiliations:** Parkersburg, W. Va.


### REPERTORY OF THE BACK.

Dr. E. H. Wilsey, Parkersburg, W. Va.

**Bruised** feeling in small of back, *Cham.*
— back and knees feel bruised while lying still in bed, better rising and walking about, *Puls.*
— lower portion of dorsal region, especially left, feels bruised, *Apis.*
— muscles of back lame and bruised, as after severe physical exertion, Vib-op.
— muscles of back feel as if bruised and will not obey the will, *Gels.*
— back and lower limbs feel as if bruised in morning on rising, Stann.
— feels as if bruised in small of back when he lies quietly upon it or sits still, better by moving, *Rhus.*
— sharp pressure and pain, as if bruised in small of back and lumbar vertebræ, especially in sacro-iliac symphsis extending into lower limbs, Hepar.
— pain in small of back while sitting at 6 p. m., Hyos.
— numb sensation in small of back, as if bruised or broken, Ox-a.
— pain while lying on back, Hyos.
— pain in left side of back, Papaya-v.
— pain in left side of back, as from much stooping on motion, worse in open air, Merc.
— pain on sides of back, as if bruised, Carbo-v.
— pain in back as if sore or bruised, Sul-ac.
— pain in back while at rest, not when in motion, *Kali-c.*
— a severe pain in back during menses, Mag-m.
— pain in the whole back after previous shooting pain in hip, Nitrum.
— lying on back caused bruised pain in tumor on left scapula, Thuja.

**Bruised** pain here and there in the back, Drosera.
— pain in whole back on and between scapula, as after long stooping, Cepa.
— pain in small of back, Coral, Clem.
— pain in small of back not worse by motion, *Cina.*
— pain in small of the back and lower limbs in evening, *Coloc.*
— pain in small of back, as if bruised or broken, worse by pressure, Plat.
— pain, as if bruised above and in small of back and both hips, with sensitiveness of parts to touch during menses, *Mag-m.*
— pain in small of back, with pain in groins when sitting or standing, better when walking, Mag-sul.
— pain as from being bruised in back and chest, Calc-c.
— pain in back while walking in open air, Zinc.
— pain in small of back, so violent as if bruised also in coccyx, *Sul.*
— violent bruised pain in small of back, Zinc.
— or sprained pain in small of back, Gambog.
— pain in back for several days, at a time also in evening after lying down, extending into nape; or at night awaking her from sleep, and so violent she dare not turn over, Nat-c.
— pain in back, *Arn.*, Ars., Asar.
— pain in small of back with weak sensation, *Ars.*
— sensation of small of back and thighs, *Hepar.*
— feeling in small of back while standing, Agari.
— sensation in back and sacrum, worse from stooping and when touched, Stront.
— pain in small of back, worse stooping, also when walking, *Meny.*
— pain in small of back from morning till evening, better by going to bed, Nat-sul.

**Bruises**, pain as from bruises in anterior part of chest and on back, Con.

**Bubbling** sensation in muscles of back and arms, Petros, Squilla.

**Burning** pain in back while lying quietly upon it, *Ars.*

— pain severe and itching constantly on back, Mag-m.

— pain with sticking in loins, coldness in back, shoulders, and arms, Hyper.

— drawing pain in small of back during sleep in morning; also a sensation of falling asleep in shoulders, disturbing sleep, disappearing on awaking, Zinc.

— tearing, drawing, or lacerating pain in back, *Nux-v.*

— severe burning pain in back, Nit-ac.

pain in back after taking cold, *Nit-ac.*

— pain in back, Carduus.

— pain in back, as from posterior walls of stomach in P. M., Lob-i.

— and drawing in back and small of back, Zinc.

— pressure upon spine somewhat above small of back, Zinc.

— in left side of upper part of back, Carbo-v.

— and pressure in back, better by walking, worse by sitting and by lying down, Nitrum.

— drawing up back from coccyx apparently under the skin, Mur-ac.

— itching on the face, on back, and on the head, Kali-c.

— in lower half of body from small of back to pit of stomach downward, Phos-ac.

— in back while walking in open air when he gets warm, Sil.

— fine shooting in small spot in middle of back, Stann.

— with slight chilliness of back there is burning of the head, face, and ears with redness of cheeks, Dig.

— sticking and tension in small of back, Ver-a.

— in back near last rib right side like heartburn, Lyss.

— stitches tearing in small of back, *Mag-m.*

— in back, Clem., Chelid., Bar-c., Lyco., *Ars.*, Ant-t.

— heat in left chest through to back, with cough, Oleum-j.

— like prickly heat in back, Apis.

**Burning** along left side of vertebræ, Asa.
— from small of back and sacrum to between scapula, Thuja.
— and biting on back, Sul.
— sensation here and there in the skin of back and arms, Lyco.
— and shooting in back in morning, ceasing after rising, but the back remains sensitive, and as if bruised, Nat-c.
— and still more shooting in the whole back, seemingly in marrow, Mag-m.
— on skin of whole back, as if he was sitting by a hot stove, with sweat on face and moderate heat, Dulc.
— on back, especially small of back several times a day, Amm-c.
— on small of back, especially during a delay of menses, Phos.
— pressure in small of back at 10 P. M., Cepa.
— in a small spot on back, *Nit-a.*
— pain in a spot about small of back, Phos-a.
— in small of back, *Phos.*
— in a small spot in small of back, better from rubbing, Phos.
— tired aching feeling and some burning in back and legs of women, *Pic-a.*
— in back before menses, *Kreos.*
— and itching in back, Agari, Daphne.
— and urging in anus with aching in small of back, Merc-per.
— and tearing in small of back, Cup.
— weariness in small of back, Populus-c.
— continuous in small of back, Kreos.
— sore denuded sensation in left side when sitting, with burning intermittent sticking, Plat.

**Burrowing** in right dorsal muscle during respiration, Stann.

**Burst** pain in small of back with hard stool and colic, as if intestines would burst, ▌▌ Lyc.

**Calves,** pain in small of back and calves, Jambos.

**Carbuncle** on back, *Hepar.*, *Lach.*, Tarant.
— on back with terrible smelling pus and blood poisoning by absorption of pus, *Anthrax*.
**Carriage**, unable to ride in a carriage from backache, Ustil.
**Catch** sudden or "kink" in back, *Secale*.
**Catching** pain in back and diaphragm, with gasping, cannot breathe without pain, Petros.
**Chair**, stiffness of back, can hardly rise from chair, *Bar-c*.
— painful stiffness of back and sacrum, especially when rising from a chair, *Caustic*.
**Change** of life, aching pain in small of back with women at, *Hydras*.
**Changes**, itching on back on scratching it changes into a pain, Kali-c.
**Changing**, severe pressive pain, changing now in chest now in the back and again in both at same time, Ant-cr.
— pain now in sacrum now in back, knees very tired; worse during rest, *Nux-m*.
**Chattering**, intense fever commencing with chills running down back like streams of cold water, causing shivering and chattering of teeth, Variol.
**Cheeks**, shivers all over back, with red cheeks and cold hands, Euphorb.
**Chest**, shooting stitches in back through to chest, *Bry*.
— slight rattling in chest at night when lying on back, Kali-c.
— shooting and stitches in back at times on right side of chest in the evening, also at night, disturbing the sleep, Nat-c.
— on lying still outstretched on back his chest feels somewhat easier, Borax.
— occasional stitch from middle of back through left side of abdomen toward chest, Kali-c.
— shooting in chest and muscles of back, Sul.
— stitches from the depths of the chest going out at the back, Caust.
— heaviness in back and tightness in chest, Carbo-v.

**Chest,** at night when lying on his back he starts up and feels a stitch in the right side of his chest, Nit-ac.

— shivering and shuddering in the back chest and epigastrium, Mez.

— sprained pain in back, arms, and chest in the A. M., Petrol.

— could not sleep during the whole menstruation on account of tearing in back, chills and heat with thirst and painful constrictions of chest, *Sepia.*

— pain through back and chest on inspiration, *Inula.*

— dull pain in back on each side about six inches above kidneys, extending through chest, Fer-iod.

— sharp pains shooting through body toward back and upward into chest with sensation of rush of blood to chest, *Cactus.*

— sharp pain extending from stomach and chest through to back, between shoulders, worse on right side, Codein.

— sticking pain in back and across chest, *Puls.*

— pain goes up with stitches through chest and settles as stinging pain under left shoulder blade, increased by deep breathing at 7 P. M., Zing.

— sprained between scapula and fore part of chest on moving arms, Carbo-i.

— stitches in right side of back and extending through to chest, Kali-c.

— sweat on back and chest while at rest during the day, Petrol.

— tearing in back and chest on motion, worse at night with sticking, Nit-a.

— soreness from back to chest, Oleum-j.

— inward pressure in back and chest up to neck, muscles and vessels seem contracted, Opi.

— stitch in back, sometimes as far as left side of chest evening and night, Nat-c.

— when breathing frequent stitches in back toward chest, Psor.

**Chest,** chronic rheumatism of chest and back, Kali-iod.
— stitches in back through into chest on least motion, *Sarsa.*

**Chill** runs continually up her back from sacrum without subsequent heat or thirst, Sul.
— severe heat in face toward evening with chill over back and hairy scalp, Sul.
— transient chill on arms, chest, and back, Sul.
— feverish chill and shuddering in back, P. M., Guiac.
— chill extending up back in evening for an hour without subsequent heat, Sul.
— in evening with a cold thrill over back, ceasing on lying down, Nitr.
— severe tearing and pricking pressure in back with a chill, later passing over into a dull pressive headache with heat in head, Sil.
— from 6 to 7 P. M. with icy cold feeling on back, so that she found it hard to get warm, Mur-a.
— pain in back during chill, Ign.
— pain in back above hips with nausea and chill, Coloc.
— pain in back before and during a chill, *Eup-per.*
— with sharp pains in back, *Con.*
— on back and small of back, Spong.
— on back for several days, Lyco.
— over whole back, Asimina, Plat.
— with backache, *Ant-t.*
— spreads from back, *Arg-m.*
— down back every P. M. from 4 o'clock till going to sleep, Mag-c.
— in back in P. M. at 3, still worse in evening, after laying down for a quarter of an hour, with cold feet, without any heat or sweat following, Lyco.
— and shudders in back without thirst, Nat-m.
— runs up back by day, Phos.
— and shuddering over back, Phos.
— beginning in small of back, *Lach.*

